# Massive Dilation of the Cystic Duct Stump With a Retained Stone Two Years After Complete Cholecystectomy Managed With Robotic-Assisted Resection

**DOI:** 10.7759/cureus.116259

**Published:** 2026-09-15

**Authors:** Mason A Johnson, Jessica E Echevarria, Christopher D Johnson

**Affiliations:** 1 General Surgery, Alabama College of Osteopathic Medicine, Dothan, USA; 2 General Surgery, Ascension Sacred Heart Bay, Panama City, USA

**Keywords:** biliary colic pain, ct (computed tomography) imaging, cystic duct dilatation, da vinci xi® system, endoscopic retrograde cholangiopancreatography (ercp), gallstone cholecystitis, postcholecystectomy syndrome, robotic surgical procedures, rural setting

## Abstract

Retained stones within the cystic duct stump are an uncommon but clinically important cause of postcholecystectomy syndrome. Dilation of the remnant duct secondary to an occluding stone is exceedingly rare, with only isolated reports of massive dilation in the literature.

We describe a 67-year-old male (BMI 31.35) who presented two years after laparoscopic cholecystectomy with recurrent right upper quadrant biliary colic. Cross-sectional imaging demonstrated progressive enlargement of the cystic duct stump from mild dilation at 241 days to marked dilation of greater than 40 mm containing a 9 mm calculus by two years and 122 days. Magnetic resonance cholangiopancreatography was contraindicated because of unknown elbow orthopedic hardware. Endoscopic ultrasound confirmed the dilated cystic duct stump and occlusive stone; however, extraction was not possible because SpyGlass cholangioscopy was unavailable. The patient therefore elected for robotic-assisted resection using the da Vinci Xi platform. The dilated remnant containing the stone was excised using intraoperative near-infrared fluorescence imaging. Histopathology confirmed benign cystic duct tissue with a 9 mm calculus and no neoplasm. The patient experienced immediate symptom resolution, was discharged on postoperative day 2, and remained asymptomatic at follow-up.

This case illustrates the mucopyocele-like pathophysiology of an obstructed cystic duct remnant. It highlights the diagnostic value of a failed therapeutic endoscopic retrograde cholangiopancreatography (ERCP) and endoscopic ultrasound, as well as the safety and precision of robotic resection when advanced endoscopic techniques are unavailable.

Retained stones in the cystic duct stump can produce progressive, massive mucopyocele-like dilation years after laparoscopic cholecystectomy, closely mimicking recurrent biliary pathology. When advanced endoscopic techniques such as SpyGlass cholangioscopy are unavailable, a failed therapeutic ERCP and endoscopic ultrasound can still provide definitive diagnosis, while robotic-assisted resection offers safe, precise treatment with immediate and sustained symptom resolution. Greater recognition of this rare entity can prevent diagnostic delay and guide individualized management in similar atypical presentations of postcholecystectomy syndrome.

## Introduction

The incidence of retained gallstones after laparoscopic cholecystectomy is estimated at 0.02% [1]. Retained stones within the cystic duct stump, although uncommon, are clinically important because they can cause progressive dilation of the remnant duct. The cystic duct stump is typically short and narrow; however, an occluding stone may lead to marked dilation that presents months to years after the index operation and closely mimics primary biliary tract disease. Dilation of the cystic duct stump secondary to a retained stone is exceedingly rare. Existing literature consists of a single case series documenting stump dilation and a 1999 case report describing massive dilation [2,3]. Diagnosis generally relies on magnetic resonance cholangiopancreatography (MRCP) or endoscopic retrograde cholangiopancreatography (ERCP), yet limitations in image quality or access to advanced endoscopic techniques can delay or complicate management. Therapeutic options remain divided: a recent case series reported six patients managed endoscopically and six managed surgically [2]. The sole case of massive dilation required surgical resection [3]. Because of the paucity of published experience, both diagnosis and optimal management of this entity remain poorly defined.

## Case presentation

A 67-year-old male weighing 104.4 kg (BMI 31.35) with a history of laparoscopic cholecystectomy performed two years prior presented with sudden-onset right upper quadrant abdominal pain identical to the biliary colic he experienced prior to his original operation. Initial imaging was consistent with acute cholecystitis and cholestasis (Figure 1). Incidental imaging obtained during evaluation for a pulmonary embolism on day 241 showed mild dilation (Figure 2). Review of the prior operative and pathology reports confirmed complete gallbladder removal without complications, thereby excluding subtotal cholecystectomy. Laboratory evaluation revealed leukocytosis with neutrophilic predominance; otherwise, results were unremarkable (Table 1).

**Figure 1:**
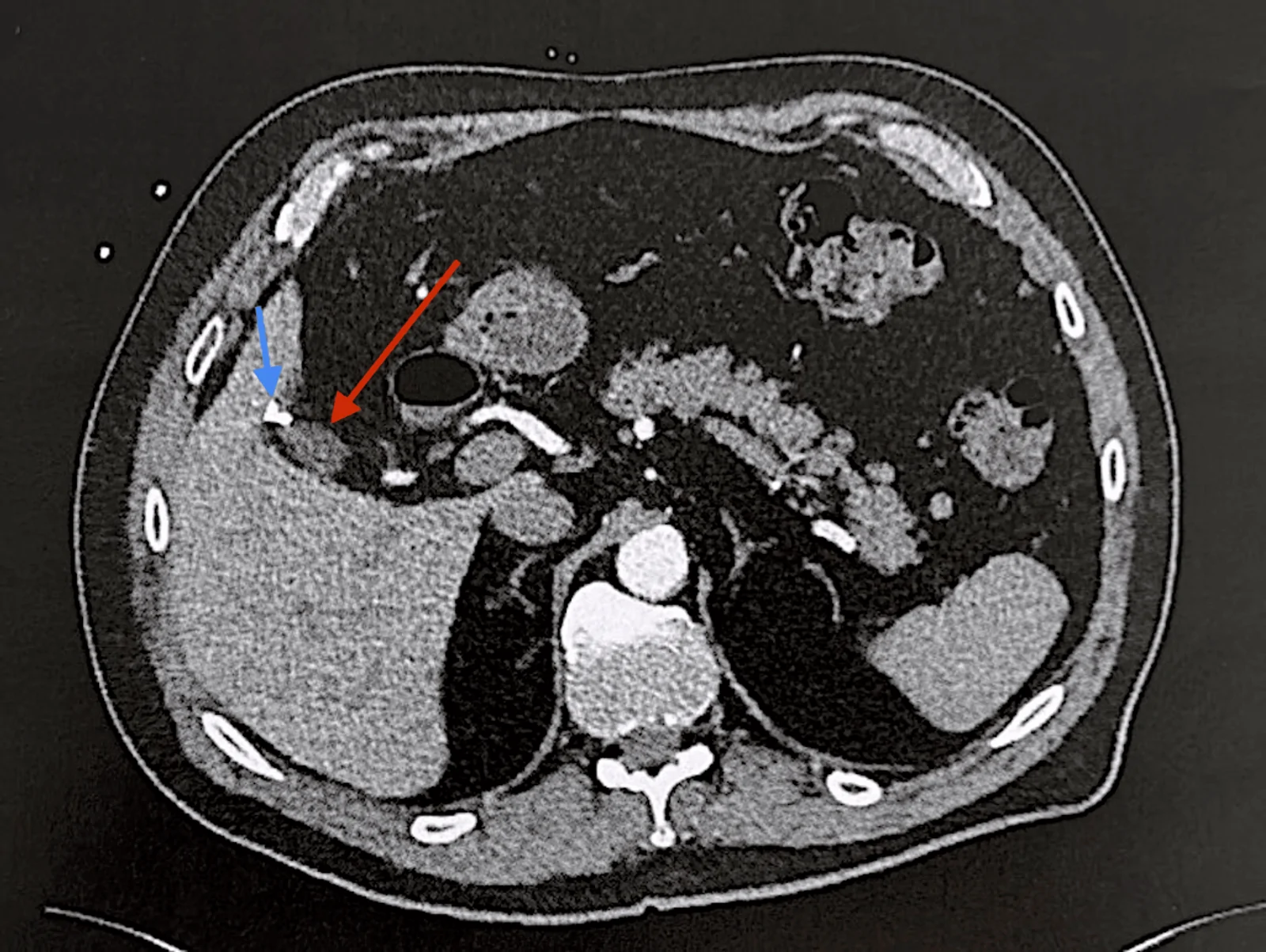
Incidental CTA of the upper abdomen on day 241 CTA demonstrating mild cystic duct remnant dilation (red arrow). Surgical clips from the index operation (blue arrow). CTA, computed tomography angiography

**Figure 2:**
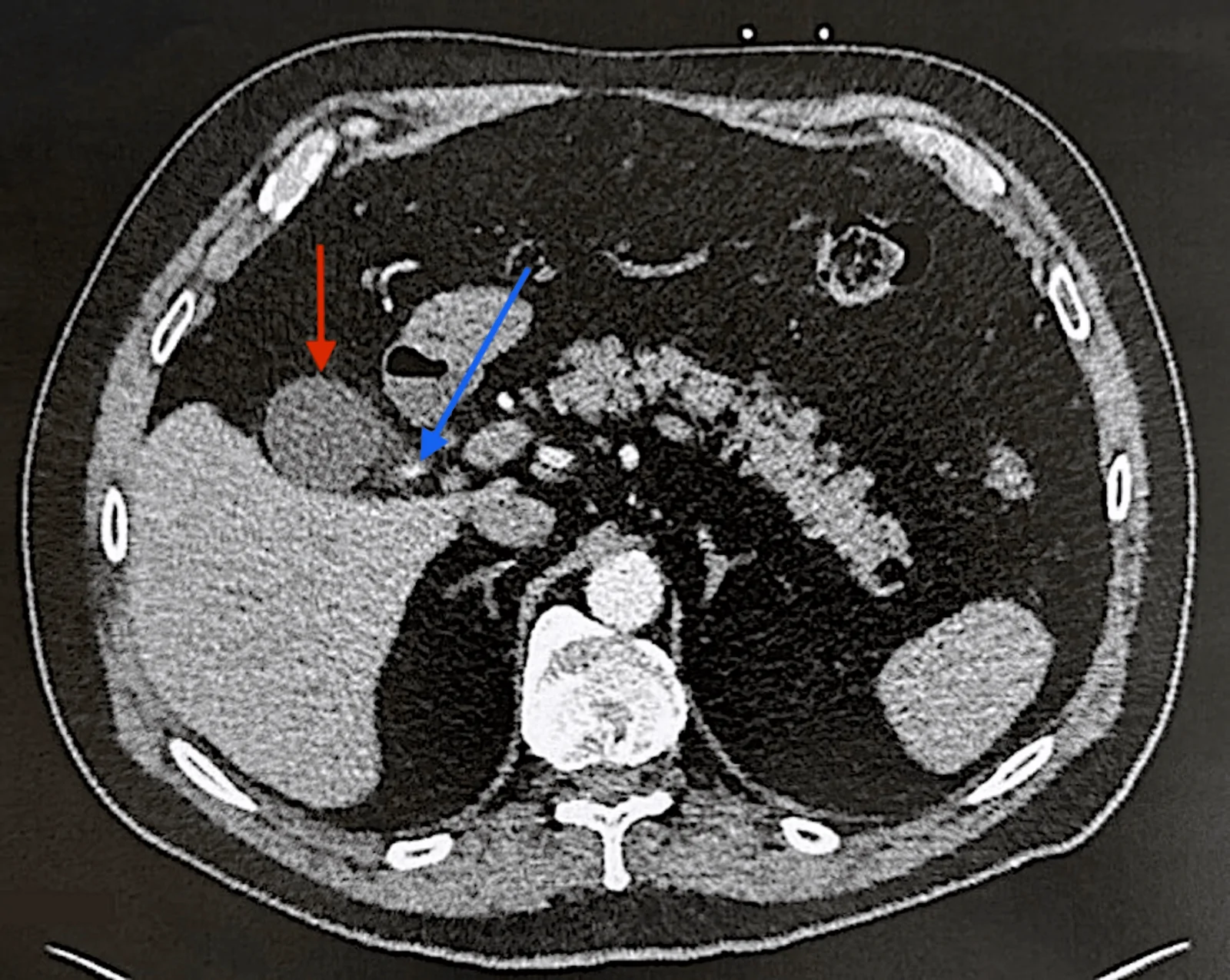
Contrast-enhanced CT of the abdomen and pelvis at presentation, two years and 122 days after surgery Contrast-enhanced CT showing significant dilation of the cystic duct stump (red arrow). Cholelith in the proximal duct (blue arrow).

**Table 1:** Patient laboratory values ^^^Value above the reference range. ^v^Value below the reference range. Hgb, hemoglobin; Hct, hematocrit; MCV, mean corpuscular volume; MCH, mean corpuscular hemoglobin; MCHC, mean corpuscular hemoglobin concentration; RDW, red cell distribution width; CO₂, carbon dioxide/serum bicarbonate; AGAP, anion gap; BUN, blood urea nitrogen; eGFR, estimated glomerular filtration rate; MPV, mean platelet volume; ALT, alanine aminotransferase; AST, aspartate aminotransferase; NRBC, nucleated red blood cells

Parameters	Patient values	Reference
WBC	12.8^^^	(4.0-11.0)x10^3^ K/uL
RBC	4.46	4.20-5.60 M/uL
Hgb	15.6	13.7-17.0 g/dL
Hct	43.5	40.0%-50.0%
MCV	97.5	81.0-98.0 fL
MCH	35.0^^^	27.0-33.0 pg
MCHC	35.9	32.0-37.0 g/dL
RDW	11.6	11.5%-14.5%
Platelet	205	150-400 K/uL
MPV	11.2	9.4-12.4 fL
Neutrophils	76.4	%
Lymphocytes	16.3	%
Monocytes	5.4	%
Eosinophils	0.9	%
Basophils	0.6	%
Imm Gran auto	0.4	%
Neutro absolute	9.82^^^	1.60-8.50 K/uL
Lymph absolute	2.09	0.50-4.80 K/uL
Mono absolute	0.70	0.00-0.80 K/uL
Eos absolute	0.11	0.00-0.70 K/uL
Baso absolute	0.08	0.00-0.10 K/uL
Imm Gran absolute	0.05	K/uL
NRBC auto	0.0	0.0%-0.0% total
NRBC Abs	0.0	x10^3^/mcL
Sodium	139	136-145 mmol/L
Potassium	4.1	3.5-5.1 mmol/L
Chloride	110^^^	98-107 mmol/L
CO_2 _	19^v^	20-30 mmol/L
AGAP	10.0	mmol/L
Bun/creat ratio	20	7-20
BUN	22.0	8.0-26.0 mg/dL
Glucose Lvl	125^^^	70-99 mg/dL
Creatinine Lvl	1.10	0.73-1.18 mg/dL
eGFR	74^v^	>90 mL/min/1.73m^2 ^
Calcium Lvl	9.2	8.4-10.8 mg/dL
Albumin Lvl	4.1	3.4-4.8 gm/dL
AG ratio	1.4	1.1-1.8
Total protein	7.1	6.4-8.3 mg/dL
ALT	45	11-55 Intl Units/L
AST	33	5-34 Intl Units/L
Alk phos	91	40-150 Intl Units/L
Bili total	0.6	0.2-1.2 mg/dL
Osmolality	282	275-300 mOsm/kg

MRCP was contraindicated because of the unknown MRI compatibility of the orthopedic hardware in the elbow. The patient was transferred to a facility with ERCP capability. Endoscopic ultrasound confirmed a dilated cystic duct stump >40 mm containing a 9 mm calculus; however, the stone could not be extracted because of its location and the unavailability of SpyGlass cholangioscopy. He was subsequently returned to our facility for definitive surgical management.

Intervention 

Given persistent symptoms and progressive dilation despite limited nonoperative options, the patient elected for robotic-assisted resection using the da Vinci Xi system. Intraoperative findings included dense adhesions in the reoperative field, a markedly dilated cystic duct stump, and a palpable intraluminal calculus (Figure 3). Careful adhesiolysis was performed, followed by meticulous dissection around the dilated remnant and common bile duct with the aid of intraoperative near-infrared fluorescence imaging (NIRF). The dilated structure was incised and drained of purulent fluid. The stone was milked proximally and extracted (Figure 4). The cystic duct stump was divided between two clips, and the dilated segment was removed within an endoscopic retrieval bag. Hemostasis was confirmed, and a closed-suction bulb drain was placed in the surgical bed. Estimated blood loss was 10 cc.

**Figure 3:**
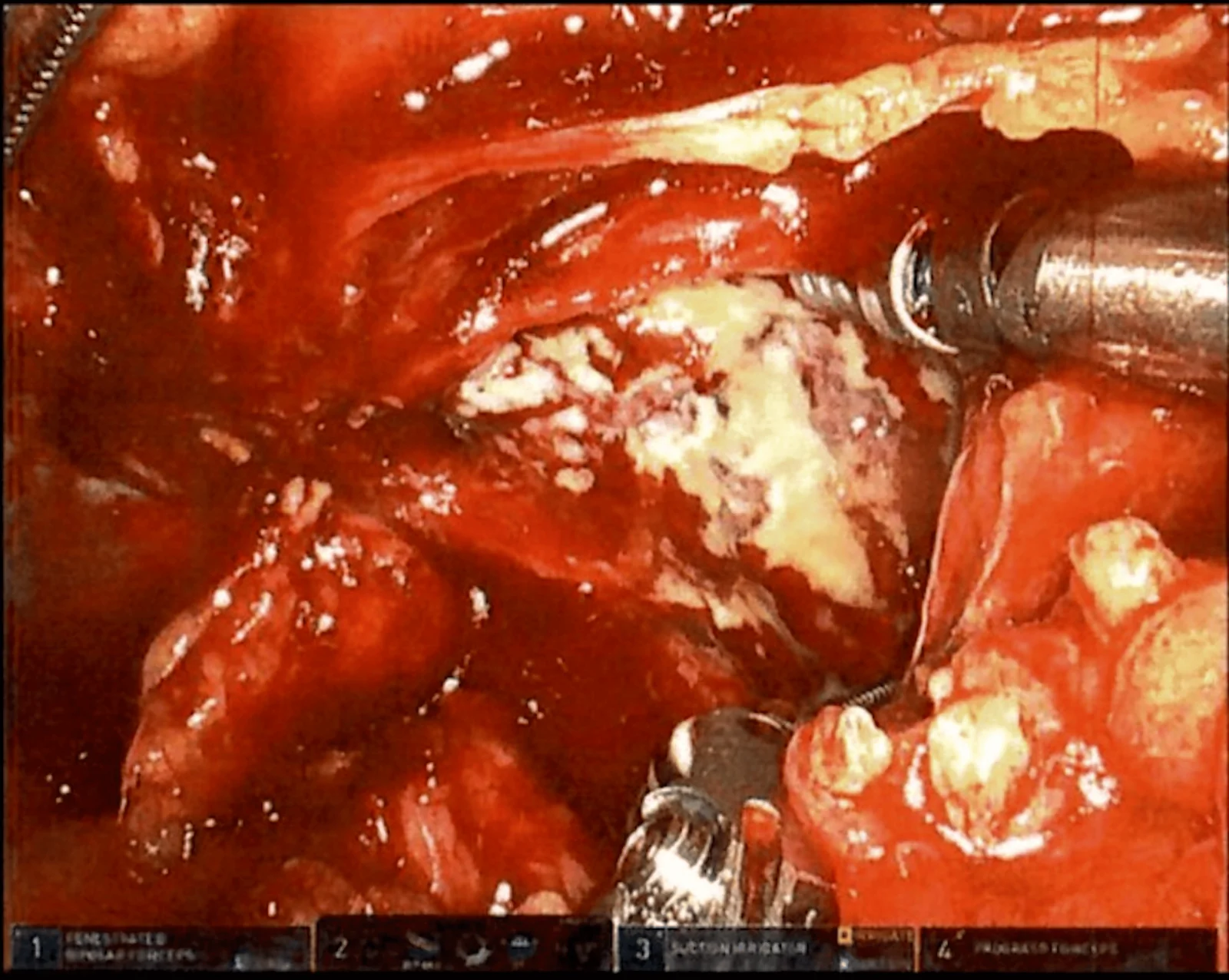
Approach to the cystic duct remnant Intraoperative view of the cystic duct stump.

**Figure 4:**
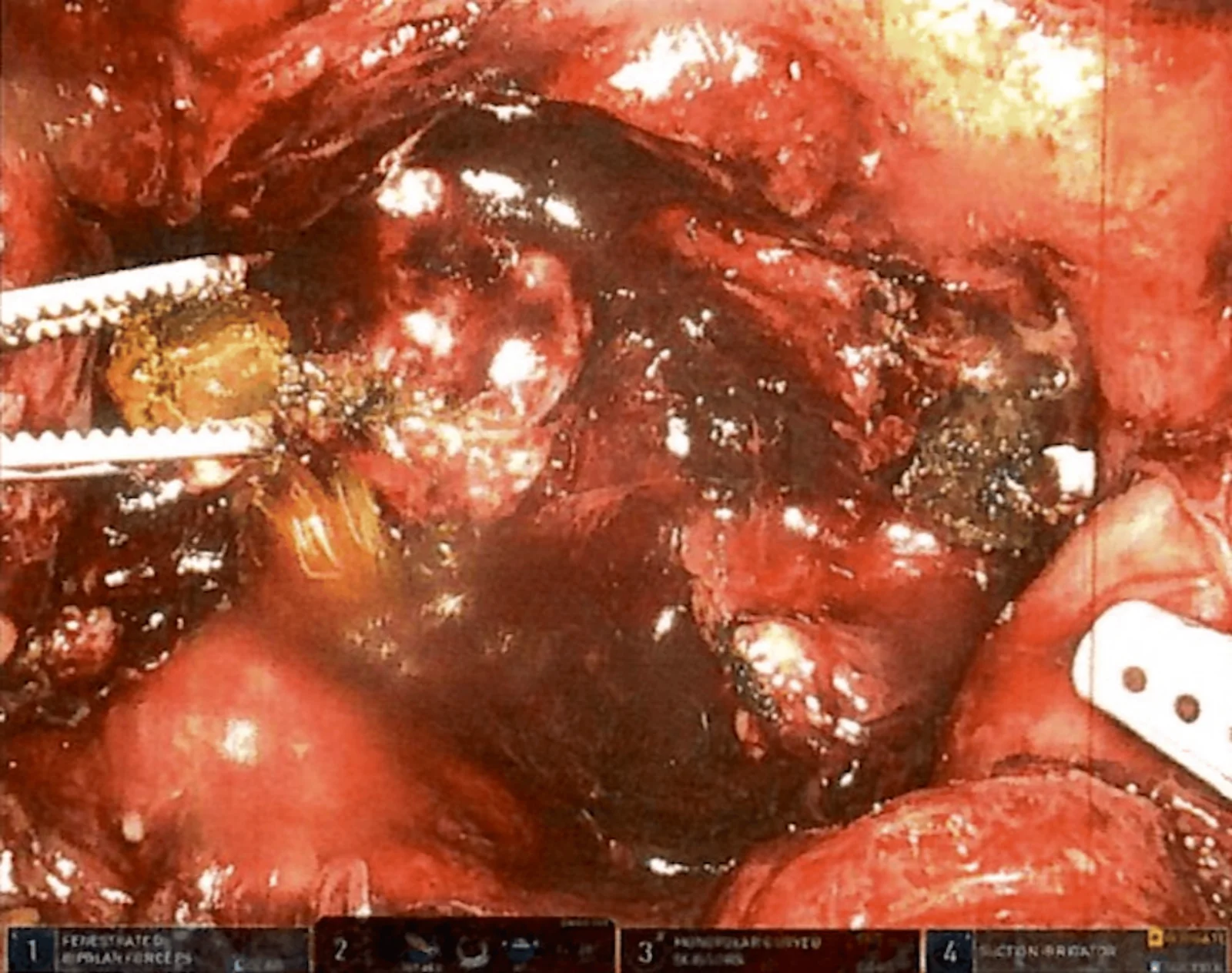
Extracted stone 9 mm stone extracted from the cystic duct stump.

Pathology 

Histopathologic examination of the resected specimen revealed a soft tissue fragment measuring 6.3 cm × 4.0 cm × 2.5 cm containing glands consistent with cystic duct tissue and two surgical clips. Dark tan/brown friable calculi measuring an aggregate of 2.1 cm × 1.5 cm × 0.7 cm were identified, with no evidence of neoplasm or lymphoid hyperplasia. No photographs were taken of the pathology slides.

Outcome 

The patient tolerated the procedure well without intraoperative complications. Right upper quadrant pain resolved immediately postoperatively. Drain output remained minimal and non-bilious. He was discharged home on postoperative day 2. The drain was removed in clinic on postoperative day 6. At the postoperative day 14 follow-up, he reported complete resolution of symptoms, with his incisions healing well.

## Discussion

Retained stones in the cystic duct stump represent a rare but recognized cause of postcholecystectomy syndrome [1]. Obstruction with subsequent dilation of the cystic duct stump is exceedingly rare, with only isolated cases in the literature describing massive dilation [2,3]. Given the paucity of published reports, it is important for clinicians to consider this entity in any patient presenting with biliary-type symptoms after cholecystectomy.

In the absence of a standardized numeric definition for a markedly enlarged or “massively dilated” cystic duct in the literature, we created an operational definition of massive dilation in this report as cystic duct enlargement exceeding reported normal measurements by an order of magnitude. Given reported average diameters of approximately 1.8 mm without calculi and 7.72 mm± 2.29 mm with common bile duct stones, a cystic duct diameter greater than 40 mm in this case met this operational definition [4,5].

In the present case, the diagnosis was confirmed by ERCP. We propose the following mechanism for the massive dilation observed: a retained stone from the original cholecystectomy completely occluded the cystic duct orifice. The cystic duct, lined by columnar epithelium with mucous glands, continued to secrete mucus into the now-obstructed, closed-loop segment. Progressive accumulation of mucus under increasing pressure resulted in gradual expansion of the remnant, ultimately reaching a size comparable to the native gallbladder. This then developed into an infection, leading to symptoms. This process accounts for the radiographic progression from mild dilation noted incidentally on computed tomography angiography 241 days postoperatively to massive dilation at presentation after two years and 122 days.

Diagnostic evaluation mirrored that of retained cystic duct stones. MRCP offers near 100% sensitivity for detecting cystic duct calculi but was contraindicated in this patient because of unknown orthopedic hardware in the elbow from a 2012 injury [6]. ERCP, although primarily validated for common bile duct stones with a sensitivity of 83%, provided definitive diagnostic information in our case [7]. Despite cannulation failure, the cholangiogram clearly demonstrated an isolated dilated cystic duct stump containing an occlusive stone. ERCP also offers therapeutic potential; however, SpyGlass cholangioscopy was unavailable at our facility, precluding endoscopic stone extraction. Surgical resection therefore became the definitive treatment. The procedure was performed robotically using the da Vinci Xi platform, with meticulous dissection to preserve the right hepatic artery given its close proximity to the dilated stump.

This case highlights several clinically relevant points. First, retained cystic duct stump stones can produce a mucopyocele-like dilation that mimics recurrent biliary pathology years after surgery. Second, when advanced endoscopic techniques are unavailable, a failed therapeutic ERCP and endoscopic ultrasound can still yield critical diagnostic clarity. Finally, robotic-assisted resection offers a safe, precise approach for massive cystic duct stump dilation when endoscopic management is not feasible. Greater awareness of this entity and its atypical presentations will help avoid diagnostic delay and guide individualized management in this uncommon scenario.

## Conclusions

This case demonstrates that retained stones within the cystic duct stump can produce progressive, massive dilation years after laparoscopic cholecystectomy, resulting in a mucopyocele-like structure that closely mimics recurrent biliary pathology. When advanced endoscopic techniques such as SpyGlass cholangioscopy are unavailable, a failed therapeutic ERCP and endoscopic ultrasound can still provide definitive diagnostic information, while robotic-assisted resection using the da Vinci Xi platform offers a safe and precise definitive treatment option. The patient experienced immediate and sustained symptom resolution with no complications. Greater awareness of this rare entity, its unique pathophysiology, and the value of minimally invasive surgical management will help clinicians avoid diagnostic delay and individualize care in similar atypical presentations.
